# Preparation and Characterization of Temperature-Sensitive Gel Plugging Agent

**DOI:** 10.3390/gels10110742

**Published:** 2024-11-15

**Authors:** Fengbao Liu, Da Yin, Jinsheng Sun, Xiao Luo, Xianbin Huang

**Affiliations:** 1 PetroChina Tarim Oilfield Company, Korla 841000, China; 2 CNPC Engineering Technology R&D Company Limited, Beijing 102206, China; 3 School of Petroleum Engineering, China University of Petroleum (East China), Qingdao 266580, China; 4 College of Chemical and Environmental Engineering, Yangtze University, Jingzhou 434000, China

**Keywords:** temperature-sensitive gel, rheological property, plugging property, cellulose based, preparation and characterization

## Abstract

In order to use intelligent gel systems to realize deep source water control in medium and high water cut reservoirs, and also to solve the shortcomings of conventional gels, such as the high chemical dose required, large profile control radius, poor temperature resistance, shear resistance, and plugging performance, a temperature-sensitive gel based on natural cellulose was developed, and the temperature resistance, rheological performance, and plugging performance of the temperature-sensitive gel were tested and evaluated. The results show that the system can maintain a viscosity retention rate of up to 95% after high-temperature aging at 90–120 °C for 50 days. When using medium- to low-salinity calcium chloride formation water for preparation, the gelation effect is good. The rheometer oscillation frequency scanning test shows that the system gel is a strong elastic body dominated by elasticity. The core displacement experiment shows that the highest sealing rate of the system is 97%, and the breakthrough pressure can reach 2.5 MPa at this time. The microstructure of the gel system was tested by infrared, and it was found that the gel system had strong hydrogen bonding and the gel had good stability. The research results contribute to improving the recovery rate of high water cut oil reservoirs.

## 1. Introduction

At present, the situation of oil reservoirs in China is complex, and the burial of crude oil reservoirs is gradually deepening. The difficulty of development is increasing, and major oil fields are also generally entering a period of medium to high water cut. Profile control and water shutoff technology for medium and high water cut reservoirs have become the key to improving the crude oil recovery rate in major oilfields [1]. The key to profile control and water shutoff technology is to use a gel system to achieve deep source water control [2]. However, with the increase in chemical dosage, the profile control radius of the conventional gel system increases. The gel is affected by rock adsorption, shearing, and dilution of edge and bottom water in the body layer, resulting in the application effect of the conventional gel system being greatly reduced. In addition, in recent years, China’s unconventional oil and gas reservoir industry has developed rapidly, including large-scale exploitation of heavy-oil/ultra-heavy-oil reservoirs. However, heavy-oil reservoirs have the characteristics of large pores, high permeability, strong heterogeneity, and large differences in interlayer transverse and longitudinal permeability [3]. Moreover, heavy oil itself has a high viscosity and density, making it difficult to flow at room temperature. Therefore, for heavy-oil wells, the viscosity of heavy oil is mainly reduced by injecting hot steam. After many rounds of stimulation of a heavy-oil reservoir, the difference between high permeability and low permeability is obvious, resulting in a low utilization rate of hot steam. Therefore, intelligent fluids such as temperature-sensitive gel are used to plug the high permeability layer of the formation for heavy-oil reservoirs, and the method of the large-pore channel is used to adjust the reservoir suction profile, improve the working efficiency of hot steam, and improve the mining output of heavy-oil wells. Therefore, in order to improve the water plugging effect of oil and gas reservoirs, it is necessary to develop a new type of gel system.

As early as 1975, Tanaka Toyichi discovered the phase transformation of polymer network structures in the process of studying polypropylene gel, and found through experiments that the gel volume will change with the change in external inspection conditions. After Flory P J, Oster G, Hirokawa Y, Tanaka T, [4,5,6,7], a large number of researchers studied gel and established the basic theory, which led to the rapid development of intelligent polymer gel. Thermosensitive gel is a kind of intelligent polymer gel. Because its swelling ratio, viscosity, and other properties change in response to external temperature changes, it can be used for deep water plugging in unconventional oil and gas reservoirs to improve the crude oil production rate.

Cellulose, as the largest organic carbon reservoir and the most abundant renewable polymer resource in nature, has gradually been discovered by researchers in recent years as a polymer intelligent material. Usually, researchers use cellulose as a raw material to carry out derivatization and modification treatments, including oxidation [8,9], esterification [10,11,12], etherification [13], cross-linking [14,15], and graft copolymerization [16,17,18]. In recent years, the research and development of temperature-sensitive gel systems using cellulose as a raw material has also become a hot spot. In 2017, Zun et al. developed a new suspension polymerization method to synthesize temperature-sensitive gel, which has a thermal responsive water-swelling property. When working, the dry particles of temperature-sensitive gel can expand to 18 times the initial size [19]. Yang and others reviewed the research progress of intelligent polymer gel, introduced some important advances in the research of intelligent polymer gel, summarized the research progress and status quo of intelligent polymer gel technology under an external stimulation environment, and discussed its application prospects [20]. Gorgieva [21] and others added citric acid on the basis of carboxymethyl cellulose and carboxyethyl cellulose to prepare a unique temperature-sensitive gel that responds to changes in the pH and temperature of the solution. Tang et al. [22] mixed shape memory polymer (SMP) particles in the gel solution, and used the formation temperature to stimulate them to complete the morphological transformation to achieve the purpose of blocking, and addressed their respective shortcomings through this release. The composition and thermal properties of SMP and gel were characterized by infrared spectroscopy and DMA. The plugging performance and mechanism of SMP/gel composites were systematically studied. The results indicate that the addition of SMP can improve the mechanical properties of composite materials. In 2023, Chen et al. [23] studied the effects of various parameters, including different poloxamer P407 concentrations, poloxamer P407/P188 (Rui Cheng Kang Pharmaceutical Technology (Shaanxi) Co., Ltd. Xi’an, China) blend ratios, and additives, on temperature-sensitive gel. The results indicate that when P407 is at a high concentration, the gelation temperature/time shows a significant downward trend. Wang et al. [24] systematically analyzed the methods of testing and adjusting the phase change temperature of thermal responsive polymer gel based on the response mechanism of thermal responsive polymer gel, and clarified how to solve the application constraints of high temperature and high salt conditions through process optimization and material innovation, ultimately broadening the application range of thermally responsive polymer gel in oil and gas production. To sum up, the existing gel system faces the shortcomings of temperature resistance, salt resistance, and environmental protection. In view of the above shortcomings, this paper introduces inorganic materials to improve the system’s temperature resistance and salt resistance, and biomass materials to improve its environmental protection performance, thus forming a gel system with a better plugging effect.

In recent years, as a new intelligent material, temperature-sensitive gel has been used in oil and gas drilling for plugging and leak prevention, polymer flooding, oil field thermal recovery channeling sealing, profile control, and water plugging. However, its application is still in its infancy. The synthesis of temperature-sensitive gel with high temperature resistance, shear resistance, good thermal stability, and good plugging effect is of great significance to the development and application of oil field development.

## 2. Results and Discussion

### 2.1. Infrared Test Results

**Figure 1:**
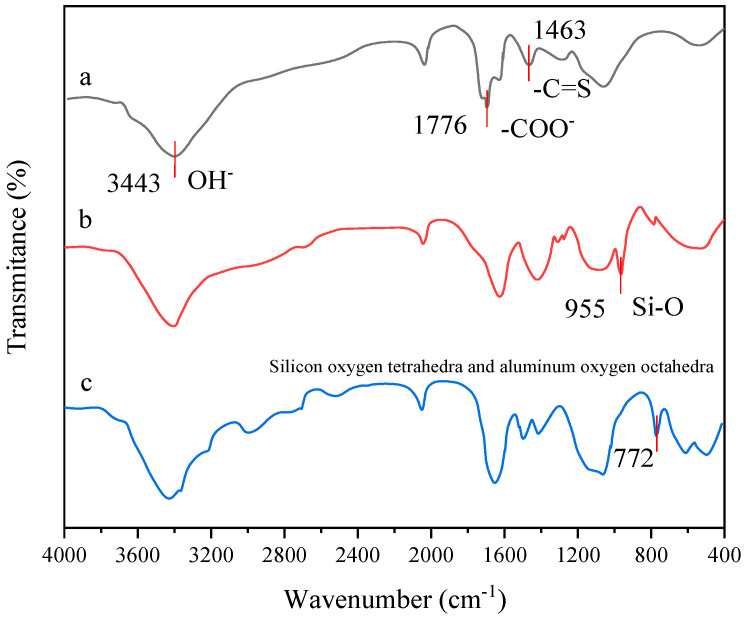
Infrared spectra of three substances. (a) Thermosensitive gel polymer cellulose A; (b) TSG-1; (c) TSG-2.

The Fourier transform infrared spectra of the three systems TSG-1, TSG-2, and thermosensitive gel polymer cellulose A were tested according to the test method in Section 4.3.1. The presence of characteristic functional groups capable of forming hydrogen bonds within the molecular structure of thermosensitive gel polymers was determined by analyzing the position of the stretching vibration peaks in the three materials. The experimental results are shown in Figure 1.

Figure 1 shows that temperature-sensitive gel polymer cellulose A contains a hydroxyl characteristic peak at 3433 cm^−1^, an ester-based characteristic peak at 1694 cm^−1^, and a C=S stretching vibration absorption peak of thiourea at 1463 cm^−1^. Compared with temperature-sensitive gel polymer cellulose A, TSG-1 is a product doped with nano silica, and there is a stretching vibration peak of a silicon oxygen bond at 955 cm^−1^. TSG-2 is a product doped with montmorillonite, which contains internal vibration peaks of silicon oxygen tetrahedra and aluminum oxygen octahedra at 772 cm^−1^.

### 2.2. Surface Tension Test Results

**Figure 2:**
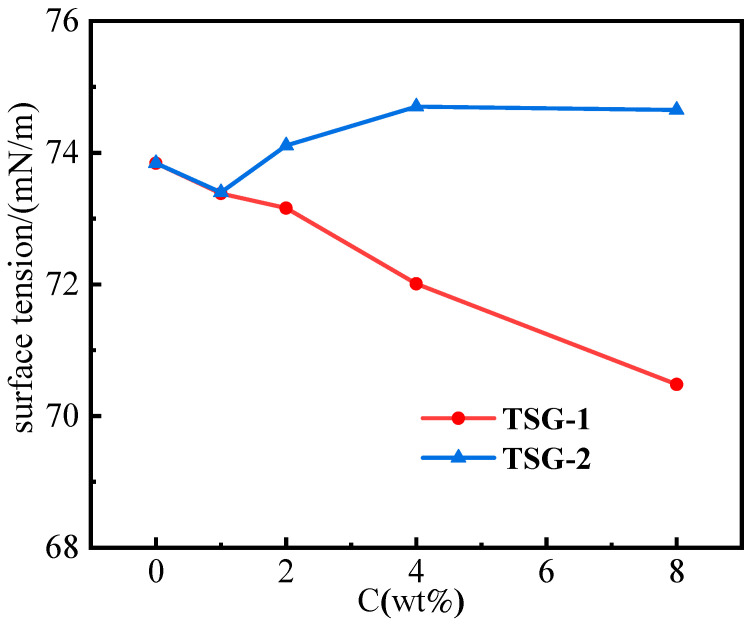
Surface tension of TSG-1 and TSG-2 in ultrapure water.

The Fourier transform infrared spectra of TSG-1 and TSG-2 were tested according to the test method in Section 4.3.1. The materials’ strong hydrogen bonding effect was determined by analyzing the relationship between surface tension and material concentration in the aqueous solutions of the two materials. The experimental results are shown in Figure 2.

Figure 2 reveals the concentration dependence of surface tension for the aqueous solutions containing TSG-1 and TSG-2. The surface tension exponentially changes as the concentration changes. The more intense the hydrogen bond action, the more prominent the decline in the surface tension of water. The surface tension values of solutions with the concentration of 0 wt.% are all 63.84 mN/m. As the agent concentration increases, there is a significant decrease in the tension of TSG-1, implying the synthesized polymer favors the formation of hydrogen bonds with water. By contrast, the tension values even rise for TSG-2 due to its sharp rise in viscosity. Through concentration, the intensity of the hydrogen bond contacts the surface tension, which achieves semi-quantitative analysis between macroscopic and microstructure phenomena.

### 2.3. Temperature Resistance Performance

Temperature-sensitive gel is a colorless, transparent, and low viscosity fluid at room temperature. When the external temperature rises to the gel temperature, the viscosity rapidly increases, forming a high-viscosity fluid. However, if the external temperature continues to rise, the apparent viscosity and gel strength of temperature-sensitive gel may decrease.

**Figure 3:**
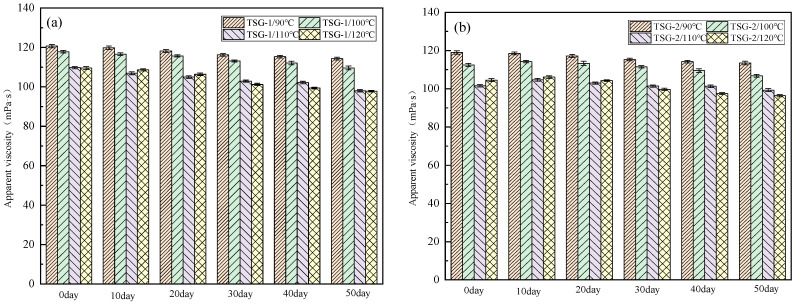
Apparent viscosity change of temperature-sensitive gel system during high-temperature aging. (**a**) TSG-1; (**b**) TSG-2.

In order to study the anti-aging performance of the temperature-sensitive gel system at high temperature, after aging at four temperature points of 90 °C, 100 °C, 110 °C, and 120 °C for a period of time, the HAAKE MARS III rheometer was used to test the apparent viscosity change of temperature-sensitive gel. The experimental results are shown in Figure 3.

It can be seen from the analysis of Figure 2 that the apparent viscosity of TSG-1 and TSG-2 systems decreases with aging time. After 50 days of aging at the above four temperature points, the viscosity retention rates of the two systems are 94.15%, 93.52%, 93.02%, and 90.16%, and 93.84%, 95.16%, 92.46%, and 91.31%, respectively. Even after 50 days of aging at 120 °C, the viscosity retention rates of the two systems can still reach about 90%. It can be seen from this that the temperature-sensitive gel has good resistance to high-temperature aging. When the temperature exceeds 100 °C, cross-linking or rearrangement of molecular chains may occur in a short period of time, leading to an increase in viscosity. However, when the time is long enough, molecular chains may break, resulting in a significant decrease in viscosity.

**Figure 4:**
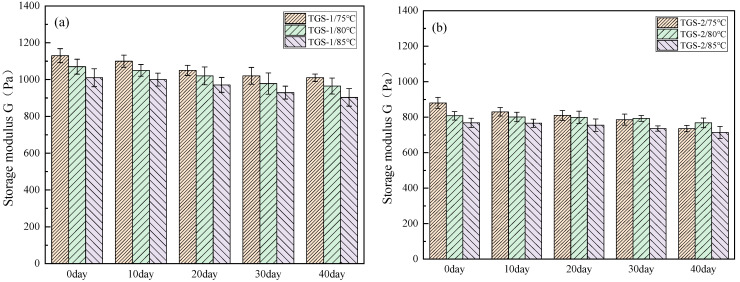
Change in storage modulus G of temperature-sensitive gel system with time. (**a**) TSG-1; (**b**) TSG-2.

Long-term thermal stability is of great significance for temperature-sensitive gel systems to realize deep source water control in oilfield application. In order to study the thermal stability of temperature-sensitive gel systems at high temperature, the three temperature points used in the test were 70 °C, 80 °C, and 85 °C. Since the strength of gel can be expressed by the storage modulus G of gel, the storage modulus of temperature-sensitive gel can be measured by a HAAKE MARS III rheometer. The experimental results are shown in Figure 4.

From the analysis of Figure 4, it can be seen that the storage modulus of TSG-1 and TSG-2 systems at different temperatures decreases with time. From the analysis of the whole process, it can be seen that within 40 days at 75 °C, 80 °C, and 85 °C, the gel strength retention rate can reach 83.78% at the lowest; specifically, at 80 °C, the gel retention rate can reach 90.35%. The reason for this is that the physical bond formed by the intermolecular force is easily affected by temperature, and the temperature-sensitive gel system is constructed by the intermolecular force of polymer. The increase in temperature will aggravate the thermal vibration of molecules and increase the molecular spacing, which will lead to the reduction and destruction of the intermolecular force of the temperature-sensitive gel system. At 80 °C, the system has strong association and a high water retention rate. Below this temperature, for example, 75 °C, the intermolecular association in the system is incomplete, and the gel strength is still in the rising stage. Above this temperature, the intermolecular interaction of the temperature-sensitive gel system is destroyed due to the high temperature, and the overall strength of the gel is reduced.

### 2.4. Salt Resistance Performance

The mineralization degree of formation water refers to the inorganic salt content in the formation water. The original formation water salinity is 1.2 × 10^5^ mg/L, and the influence of formation water salinity in the range from 7.5 × 10^3^ mg/L to 1.2 × 10^5^ mg/L on the salt resistance of gel was tested by dilution.

**Figure 5:**
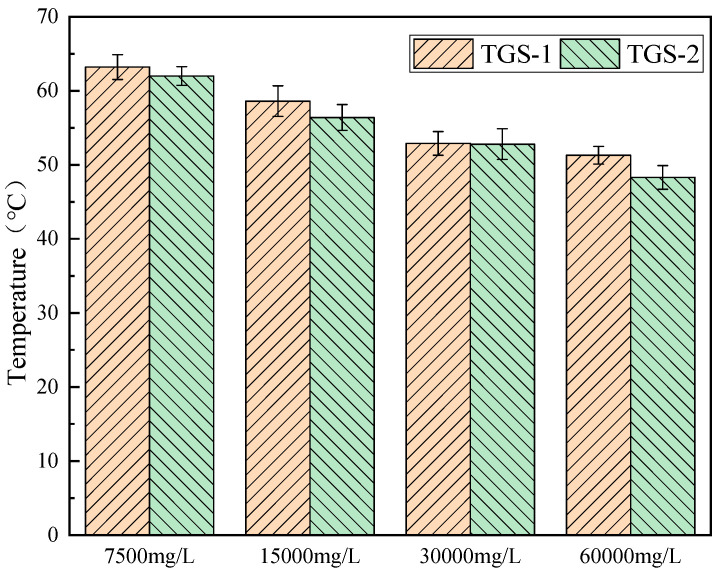
Temperature scanning rheological diagram prepared with formation water of different mineralization degrees.

In order to study the influence of the salinity of formation water on the gel temperature and gel strength of the system, the salinity of formation water was tested as the scanning rheogram of the storage modulus/loss modulus and loss coefficient of the above five configuration systems against temperature. The test results are shown in Figure 5.

The temperature at which the storage modulus G and the loss modulus G′ intersect is the gel temperature of the system [24]. It can be seen from Figure 4 that the gel temperature of the system decreases with the increase in the salinity of the formation water, because the added inorganic salt ions will compete with the polymer molecules to attract water molecules to move, destroying the ordered water solvation outer layer of the polymer, and resulting in the exposed leakage of the polymer, which causes the thermal cooperation of the system to occur at a lower temperature.

**Figure 6:**
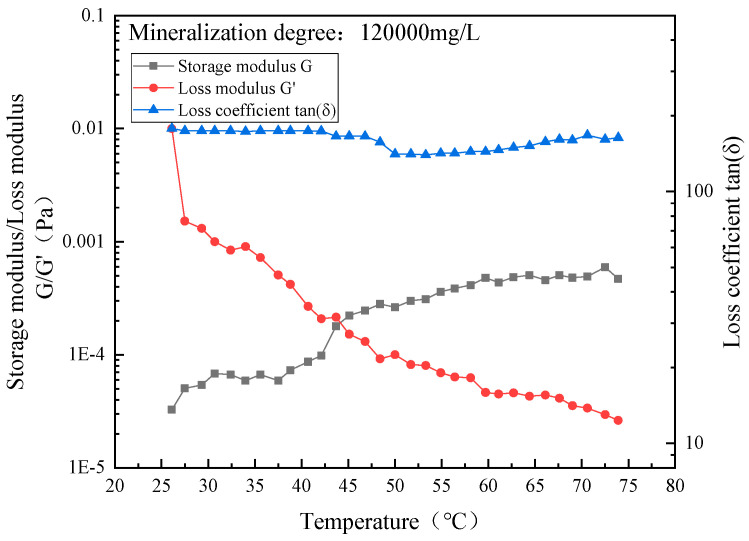
Temperature scan rheological diagram of TSG-1 system prepared with high-salinity formation water.

**Figure 7:**
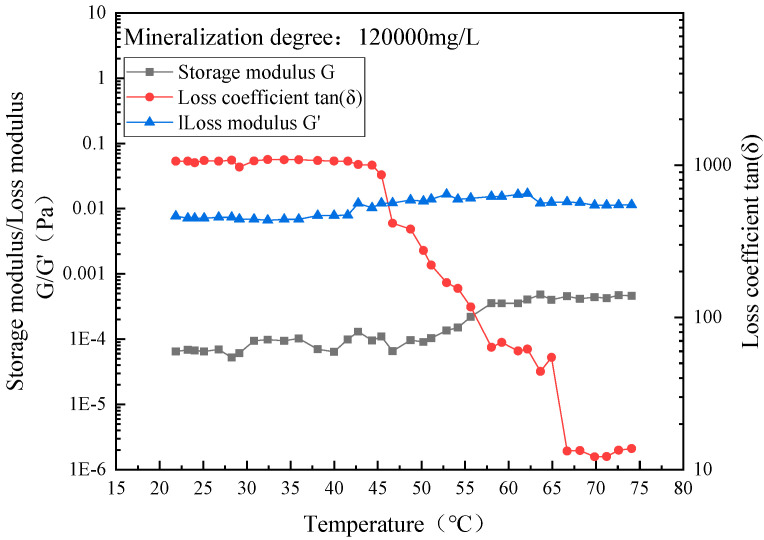
Temperature scan rheological diagram of TSG-2 system prepared with high-salinity formation water.

At high salinity (1.2 × 10^5^ mg/L), as the temperature increases, the loss modulus of the system remains basically unchanged, while the storage modulus slightly increases. There is no intersection point between the storage modulus G and the loss modulus G′ throughout the process, indicating that the system does not form a gel under high salinity. The main reason for this is that inorganic salt ions in the high-salinity formation water will interact with the polymer molecular chains, meaning the hydrophobic groups in the temperature-sensitive polymer molecules are unable to break free from the outer layer of water solvation, resulting in non-gelation of the system. The experimental results are shown in Figure 6 and Figure 7.

### 2.5. Shear Resistance Performance

**Figure 8:**
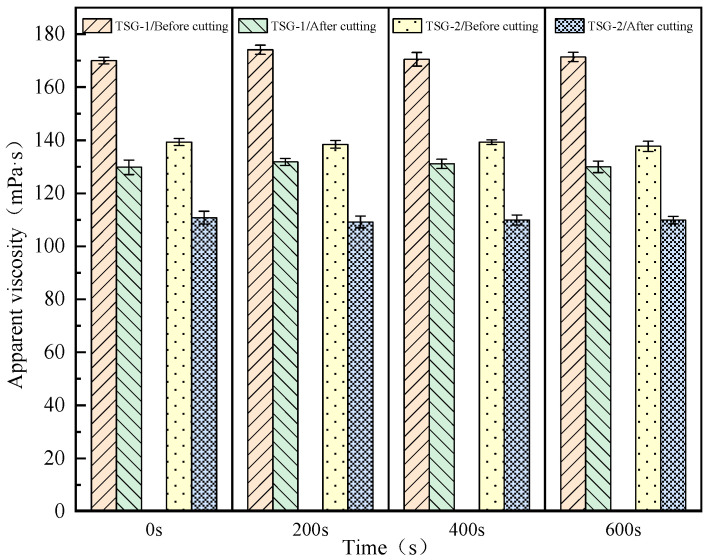
Simulated apparent viscosity change curve before and after rock shear.

The simulated core with water-test permeability of 1182 × 10^−3^ μm^2^ was used to test the change in apparent viscosity of the temperature-sensitive gel system before and after dynamic shear in the simulated core, so as to simulate the shear process of the temperature-sensitive gel system in the formation core. The experimental results before and after shear are shown in Figure 8.

It can be seen from the results in Figure 8 that the apparent viscosity of the temperature-sensitive gel system TSG-1 before shearing is 175.3 mPa·s and the apparent viscosity after simulated core shearing is 128.6 mPa·s. The apparent viscosity of TSG-2 before shear is 140.5 mPa·s, and after simulating core shear, the apparent viscosity is 106.32 mPa·s. Therefore, in general, the viscosity retention rate of the temperature-sensitive gel system after simulated core shearing is high, indicating that the shear resistance of the temperature-sensitive gel system is good.

### 2.6. Viscoelastic Performance

**Figure 9:**
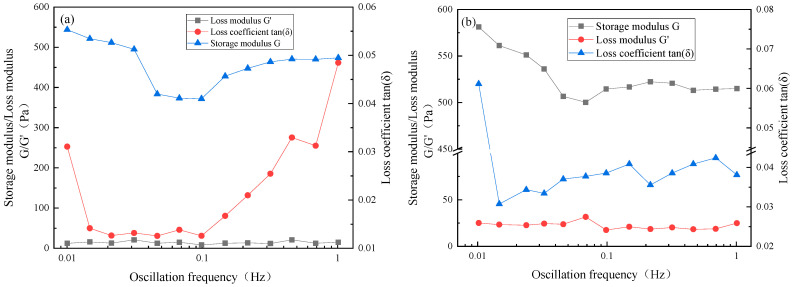
Viscoelastic test curve of temperature-sensitive gel with variable oscillation frequency. (**a**) TSG-1; (**b**) TSG-2.

Using the vibration frequency scanning unit of the rheometer, the stress is selected as 1 Pa, and the vibration frequency varies from 1 to 0.01 Hz. The viscoelastic properties of the system gel under a variable vibration frequency are tested. The test results are shown in Figure 9.

It can be seen from Figure 9 that the storage modulus G of the thermosensitive gel system decreases first and then stabilizes with the increase in the oscillation frequency, while the loss modulus G′ is basically stable, and the loss modulus is obviously smaller than the storage modulus, indicating that the thermosensitive gel system is a strong elastic body mainly guided by elasticity. When the oscillation frequency is less than 0.1 Hz, the storage modulus G′ shows a downward trend. When the oscillation frequency is greater than 0.1 Hz, the storage modulus G′ basically remains unchanged because of the destruction–reconstruction process of the gel three-dimensional network. When the gel network structure is formed, the external force will destroy the gel network, so the storage modulus G′ decreases, but the temperature-sensitive gel will also be affected by the temperature to reconstruct the network structure for thermal cooperation. Therefore, when the gel is destroyed and reconstructed in a dynamic equilibrium process, the storage modulus G′ basically remains unchanged.

### 2.7. Creep Performance

**Figure 10:**
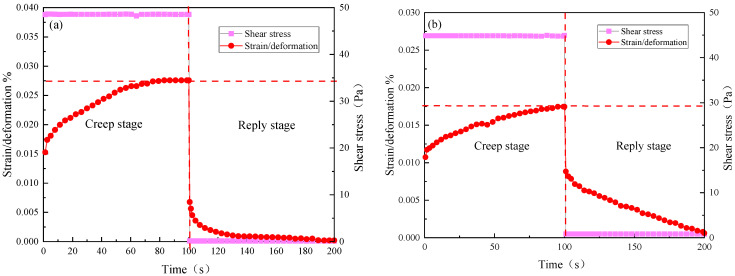
Creep recovery performance of temperature-sensitive gel system. (**a**) TSG-1; (**b**) TSG-2.

The creep performance test shows the response of material strain to time when the external stress is constant, while the recovery performance test is the time it takes for the material to recover to before elastic deformation after removing the external force [25]. Using the control stress (CS) scanning mode of the HAAKE MARS III rheometer, the applied stress value is usually less than half of the yield stress value. Therefore, a stress value of 40 Pa is set to test the viscoelastic response performance of the TSG-1 and TSG-2 systems. The experimental results are shown in Figure 10.

Figure 10a shows that when the shear stress of 40 Pa is applied to the montmorillonite-compounded TSG-1 system, the gel gradually deforms within 0~100 s. The time for gel to recover from deformation after unloading the stress is 100 s. During the creep recovery performance test, the shape of the gel system is small. As shown in Figure 9b, the TSG-2 system compounded with nano silica exhibits better strain/deformation capacity. The time for gel to recover from deformation after unloading stress is also 100 s.

### 2.8. Sealing Performance

Simulated cores were used to evaluate the plugging performance of temperature-sensitive gel.

**Figure 11:**
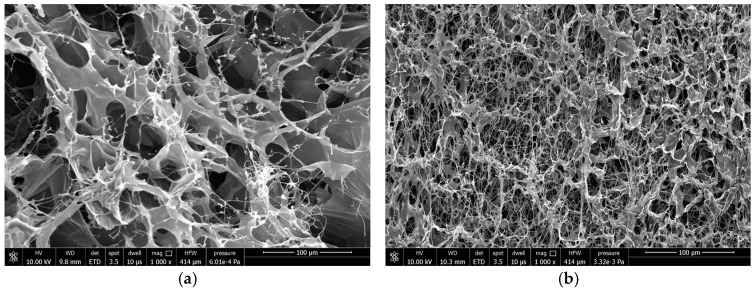
Microstructure of two gel systems. (**a**) TSG-1, (**b**) TSG-2.

The permeability before plugging of the three simulated cores was measured by the water logging permeability method as 1182 × 10^−3^ μm^2^, 2003 × 10^−3^ μm^2^, and 3319 × 10^−3^ μm^2^, respectively. Then, the core displacement device was used to carry out the dynamic plugging experiment of the TSG-2 temperature-sensitive gel system. The plugging rate was calculated by simulating the changes in core permeability before and after plugging to evaluate the effect of the temperature-sensitive gel system on formation plugging. The experimental results are shown in Table 1. The morphology of gel after plugging is shown in Figure 11.

It can be seen from Table 1 that when plugging experiments are carried out with simulated cores, the breakthrough pressure of the temperature-sensitive gel system is more than 2 MPa, and the plugging rate is more than 96%, indicating that the plugging performance of the temperature-sensitive gel system is good.

As shown in Figure 11, both gel systems have a dense network structure. For temperature-sensitive gel systems, the adhesion of the gel network structure formed by physical bond thermal association with the wall surface is limited, while the simulation core is made by pressing a certain mesh of quartz sand. Its pores are connected in multiple ways. The wall between pores is rough and has higher viscosity resistance. When the gel system is gelled to form a complex network structure, it can be better retained in the pore fracture structure. The seepage resistance of water drive is greater and the breakthrough pressure is higher, which can achieve the purpose of plugging the formation and controlling water.

## 3. Conclusions

Infrared spectroscopy was used to study the vibration peak changes in group structures of three substances, revealing strong hydrogen bonding in TSG-1 and TSG-2 temperature-sensitive polymers, thereby enhancing gel stability. The temperature-sensitive gel showed high resistance to high-temperature aging and long-term thermal stability, with viscosity and strength retention rates over 90% and 89.83%, respectively, after prolonged exposure. High salinity reduced gel temperature, preventing gelation above 1.2 × 10^5^ mg/L. The gel exhibited shear thinning but maintained over 81% viscosity post-shearing, indicating good shear resistance and strong elasticity. Core displacement tests demonstrated the gel’s plugging performance, with a maximum breakthrough pressure of 2.5 MPa and a plugging rate of 97%, confirming its effectiveness in formation plugging.

## 4. Materials and Methods

Temperature-sensitive gel is a kind of intelligent gel that can suddenly change its volume with the change in ambient temperature. In this paper, a new type of temperature-sensitive gel system is developed by comparison. This type of temperature-sensitive gel system uses cellulose A as the main agent, and nano silica and silicate crystal montmorillonite as the composite reinforcing agent, and a regulator is added to form a temperature-sensitive gel system. The high-temperature aging resistance, salt resistance, long-term thermal stability, shear resistance, viscoelastic performance, creep recovery performance, etc. of the temperature-sensitive gel system are evaluated. The indoor simulation plugging test is carried out through the core displacement device to evaluate the plugging effect of the temperature-sensitive gel system on the formation.

### 4.1. Materials

The required chemical reagents and instruments for this experiment are shown in Table 2 and Table 3. All materials were used as-is without purification or pretreatment.

### 4.2. Synthesis of Sealing Agents

Temperature-sensitive polymers have low solubility and a slow dissolution rate in cold water at room temperature, and are prone to clumping during the dissolution process, resulting in insufficient dissolution. Therefore, stirring and heating are often used for dissolution in experiments. Firstly, 0.6 g of thermosensitive polymer cellulose A powder and 0.3 g of thiourea were prepared in two identical base solutions using deionized water at room temperature in a 50 mL beaker. The solutions were heated at 45 °C for 30 min and the stirrer speed was maintained at 1200 r/min. Then, 0.3 g of montmorillonite and nano silica were weighed separately and added to the two systems to form TSG-1 (containing montmorillonite) and TSG-2 (containing nano silica) systems, respectively. Heating and stirring was continued for 1 h, and the prepared sample was allowed to mature for 24 h before use.

### 4.3. Performance Testing

#### 4.3.1. Infrared Testing

The process is as follows: Place the prepared thermosensitive gel TSG-1 and TSG-2 systems in an electric blast box, take out small thermosensitive gel samples that are stable and gelatinized, put them into a petri dish and freeze dry with liquid nitrogen, then put them into a vacuum freeze dryer to remove the vacuum. Freeze dry them for 24 h, take them out, and put them into a mortar and add an appropriate amount of potassium bromide to grind them. Put them into a powder tablet press to form discs after they are completely ground, and test the infrared spectral curve of the samples with an FTIR-650 spectrometer (resolution 1.5 cm^−1^, spectral range 400–4000 cm^−1^). Before conducting sample measurements, background measurements were taken. In order to compare the spectra obtained from different samples, the spectra were normalized to have the same reference value.

#### 4.3.2. Surface Tension Testing

Surface tension as a simple indicator was analyzed using a surface tensiometer (measured with an SDC-500 automated surface tensiometer) to examine molecular hydrogen bonding interactions. A series of TSG-1 and TSG-2 solutions at different concentrations were prepared in ultrapure water. The surface tension values were recorded and normalized under controlled conditions to account for changes in the sample and the environment, and finally the surface tension of the two gels was analyzed and recorded as a function of concentration.

#### 4.3.3. Rheological Performance Testing

The rheological property test of temperature-sensitive gel generally includes measuring its rheological behaviors at different temperatures, such as shear rate, shear stress, rheological index, and other parameters. This is helpful to understand the flow characteristics and deformation behavior of gel under different conditions, as well as its stability and controllability in practical applications.

The HAAKE MARS III rheometer produced by German Thermo Fisher Company was used to test the rheological properties of temperature-sensitive gel system, including temperature resistance (75–120 °C, 7.2 s^−1^), salt resistance (7.5 × 10^3^–1.2 × 10^5^ mg/L, 7.2 s^−1^), shear resistance (0–10 min, 7.2 s^−1^), viscoelasticity (0.01–1 Hz), and creep performance (0–100 s, 40 Pa). The high-temperature and high-pressure rheometer used is in a closed space, so the effects of sublimation, evaporation, and adsorption on viscosity have not been considered for the time being.

#### 4.3.4. Sealing Performance Test

The plugging performance of the temperature-sensitive gel system was evaluated by a core displacement device, and simulated cores were selected to simulate the plugging effect of the temperature-sensitive gel system on sandstone-type pores. To determine the plugging effect of the system, the pore volume and permeability of the simulated core should be measured before the experiment, and then the plugging rate of the temperature-sensitive gel system can be calculated.

(1) Measurement of pore volume in simulated rock cores

Firstly, place the simulated rock core in a constant temperature oven at 130 °C for 24 h to dry, then weigh m_1_. After drying, remove the simulated rock core and vacuum-extract it. After vacuum extraction for 4 h, use a rock core saturation device to saturate distilled water for 12 h and weigh m_2_. Finally, calculate the pore volume of the simulated rock core.

V = (m_2_ − m_1_)/ρ_w_ (1)

where V is the pore volume, m_1_ is the dry simulated core mass, m_2_ is the saturated simulated core mass, and ρ_w_ is the saturated distilled water density.

(2) Measurement of simulated core permeability

Firstly, place the simulated rock core (length L, cross-sectional area A) after saturated distilled water into the core gripper. Then, use an advection pump to increase the annular pressure, which should be greater than the inlet water pressure. After the outlet water flow q stabilizes, record the inlet water pressure as P_1_ and outlet water pressure as P_2_. Test the viscosity u of the injected water and calculate the permeability of the simulated rock core using Darcy’s formula.

K = quL/A(P_1_ − P_2_) (2)

(3) Determination of sealing rate

First, connect the core displacement device, set the experimental temperature of the constant temperature drying oven to 75 °C, and inject the distilled water with a horizontal flow pump at a rate of 0.2 mL/min after stabilizing for 2 h. After the flow rate of the distilled water at the outlet is stable, record the pressure at this time as P_1_. Then, in order to simulate the injection of 0.6–0.8 PV into the core, inject the prepared temperature-sensitive gel system at a rate of 0.5 mL/min and record the pressure as P_2_. Subsequently, stabilize at 75 °C for 2 h, and after the colloid stabilizes, inject distilled water using a water drive method at a rate of 0.5 mL/min and record the instantaneous breakthrough pressure value as P_3_.

η = (P_3_ − P_1_)/P_3_ (3)

## Figures and Tables

**Table 1:** Evaluation test results of core water drive sealing performance.

Number	Water Permeability Measurement/10^−3^ μm^2^		Breaking Through Pressure/KPa	Sealing Rate/%
	Before Sealing	After Sealing		
1	1182	30.32	2518	97.43
2	2003	67.23	2337	96.64
3	3319	118.45	2151	96.43

**Table 2:** Chemical reagents required for the experiment.

Materials	Type	Manufacturer
Cellulose A	Analytical purity	Sigma Aldrich Trading Co., Ltd. Shanghai China
Nano silica	Analytical purity	Shanghai McLean Biochemical Technology Co., Ltd. Shanghai China
Montmorillonite K-10	Analytical purity	Shanghai McLean Biochemical Technology Co., Ltd. Shanghai China
NaCl	Analytical purity	Shanghai McLean Biochemical Technology Co., Ltd. Shanghai China
CaCl_2_	Analytical purity	Shanghai McLean Biochemical Technology Co., Ltd. Shanghai China
Thiourea	Analytical purity	Aladdin Reagent Company. Shanghai China

**Table 3:** Instrument information required for the test.

Test Instruments	Type	Manufacturer
Digital electronic balance	YP2001	Sanfeng Precision Measuring Instrument Co., Ltd. Shanghai China
Digital constant temperature multi head magnetic stirrer	HJ-6A	Jiangsu Ronghua Instrument Manufacturing Co., Ltd. Taizhou China
Electric blast drying oven	YH-H-225L	Dongguan Yiheng Instrument Technology Co., Ltd. Dongguan China
Vacuum freeze dryer	FD-1-50	Beijing Boyikang Experimental Instrument Co., Ltd. Beijing China
Rotary vane vacuum pump	2XZ-2	Linmao Technology Co., Ltd. Beijing China
Powder tablet press	769YP-15A	Tianjin Keqi High tech Co., Ltd. Tianjing China
Fourier transform infrared spectrometer	FTIR-650	Tianjin Gangdong Technology Co., Ltd. Tianjing China
Surface tension tester	SDC-500	Foshan Huashitong Precision Instrument Co., Ltd. Foshan China
Rheometer	HAAKE MARS III	Themo Fisher. Shanghai China
Advection pump	2PB00C	Beijing Satellite Manufacturing Factory. Beijing China

## Data Availability

All data and materials are available on request from the corresponding author. The data are not publicly available due to ongoing researches using a part of the data.
